# Supplementary material for: Mechano-redox control of integrin de-adhesion
Source: eLife. 2018 Jun 22;7:e34843. doi: 10.7554/eLife.34843 (PMC6054529; doi:10.7554/eLife.34843)
Supplement: Supplementary file 1. [file elife-34843-supp1.docx]

**Supplementary File 1**. List of β3 integrin cysteine containing peptides detected by mass spectrometry.

| Disulfide bond | Cys position | Peptide sequence*† |
| --- | --- | --- |
| 5-23 | 23 | AVSPMCAW  AVSPM(oxidation)CAW |
| 13-435  16-38 | 13, 42 | GVSSCQQCL |
| 26-49 | 26  49 | CSDEALPL  ENLLKDNCAPESIEFPVSEAR  LKDNCAPESIEFPVSEAR  KDNCAPESIEFPVSEAR  DNCAPESIEFPVSEAR |
| 177-184 | 177  184 | MYISPPEALENPCY  M(oxidation)YISPPEALENPCY  ISPPEALENPCY  ISPPEALENPCYDMK  ISPPEALENPCYDM(oxidation)K  ENPCY  ENPCYDMK  TTCLPMF  TTCLPM(oxidation)F  TTCLPMFGY  TTCLPM(oxidation)FGY |
| 232-273 | 232  273 | DAPEGGFDAIMQATVCDEK  DAPEGGFDAIM(oxidation)QATVCDEK  DAIMQATVCDEK  DAIM(oxidation)QATVCDEK  LAGIVQPNDGQCHVGSDNHY  AGIVQPNDGQCHVGSDNHY |
| 374-386 | 347  386 | SLSFN(deamidated)ATCL  SFN(deamidated)ATCLNNEVIPGL  SFN(deamidated)ATCLNNEVIPGLK  N(deamidated)ATCLNNEVIPGL  N(deamidated)ATCLNNEVIPGLK  N(deamidated)ATCLN(deamidated)NEVIPGLK  KSCMGL  KSCM(oxidation)GL |
| 406-433 | 406 | GCPQEK |
| 437-457  448-460 | 457, 460  448 | ECGVCR  CNNGN(deamidated)GTF  CNN(deamidated)GN(deamidated)GTF |
| 462-471 | 462 | CGPGW |
| 473-503 | 473 | LGSQCECSEEDY |
| 486-501 | 486 | RPSQQDECSPR |
| 495-506 | 495 | EGQPVCSQR |
| 508-521  523-544 | 521, 523 | YCECDDF  CECDDF |
| 549-558  560-583 | 549  558, 560 | CDSDWTGY  YCN(deamidated)CTTR  CN(deamidated)CTTR |
| 567-581 | 567 | TDTCMSSNGL  TDTCMSSNGLL |
| 575-586 | 575 | LCSGR |
| 588-598 | 598 | GDTCEK |
| 608-655 | 655 | KDTGKDAVN(deamidated)CTY  DAVN(deamidated)CTY |
| 614-635  617-631 | 614, 617  631  635 | KKECVECK  KECVECK  DRGALHDENTCNR  DRGALHDENTCNRY  GALHDENTCNR  GALHDENTCNRY  HDENTCNRY  CRDEIESVK |
| 663-687 | 663  687 | KNEDDCVVR  KNEDDCVVRF  NEDDCVVR  SILYVVEEPECPK  YVVEEPECPK  VVEEPECPK  VVEEPECPKGPDIL  VVEEPECPKGPDILVVL |

* Cys was labelled with ^12^C-iodoacetanilide (133.05276) or ^13^C-iodoacetanilide (139.07289).

† Asn is a glycosylated residue and treatment with PNGase F resulted in its deamidation.
