# Supplementary material for: Mechano-redox control of integrin de-adhesion
Source: eLife. 2018 Jun 22;7:e34843. doi: 10.7554/eLife.34843 (PMC6054529; doi:10.7554/eLife.34843)
Supplement: Supplementary file 2. [file elife-34843-supp2.docx]

**Supplementary File 2**. Structural features of the β3 integrin Cys177-Cys184 disulfide bond.

| αIIbβ3 structures  with Ca^2+^ and Mg^2+^ | Chain | Cys177 solvent accessibility (~Å^2^) | Cys184 solvent accessibility (~Å^2^) | Configuration |
| --- | --- | --- | --- | --- |
| Bent conformation  (PDB code 3fcs) | B | 0 | 17 | ‒/+RHhook |
|  | D | 1 | 17 | ‒/+RHhook |
| Extended apo conformation  (PDB code 3fcu) | B | 0 | 11 | ‒/+RHhook |
|  | D | 0 | 12 | ‒/+RHhook |
|  | F | 0 | 12 | ‒/+RHhook |
| Extended holo conformation  (PDB code 2vdo) | B | 0 | 13 | ‒/+RHhook |
| Extended holo conformation  (PDB code 2vdp) | B | 0 | 12 | ‒/+RHhook |
| Extended holo conformation  (PDB code 2vdq) | B | 0 | 12 | ‒/+RHhook |
| Extended holo conformation  (PDB code 2vdr) | B | 1 | 12 | ‒/+RHhook |
