# Supplementary material for: Mechano-redox control of integrin de-adhesion
Source: eLife. 2018 Jun 22;7:e34843. doi: 10.7554/eLife.34843 (PMC6054529; doi:10.7554/eLife.34843)
Supplement: Supplementary file 3. — Phylogenetic tree for the integrin β-subunit Cys177-Cys184 disulfide bond. The cysteines forming the disulfide are highlighted in yellow. [file elife-34843-supp3.docx]

**Supplementary File 3**. The βI Cys177-Cys184 disulfide bond is conserved in 7 of 8 β integrins. Phylogenetic tree for the integrin β-subunit Cys177-Cys184 disulfide bond. The cysteines forming the disulfide are highlighted in yellow.
